# Management of afferent loop syndrome with a second endoscopic ultrasound-guided gastrojejunostomy in advanced cholangiocarcinoma

**DOI:** 10.1055/a-2845-0680

**Published:** 2026-04-20

**Authors:** Antonio Fuso, Andrea Lisotti, Graziella Masciangelo, Pietro Fusaroli

**Affiliations:** 1 18578 Gastroenterology Unit, Imola Hospital Santa Maria della Scaletta, Imola, Italy

Malignant gastric outlet obstruction and afferent loop syndrome are challenging complications in advanced pancreatobiliary malignancies, often precluding surgical bypass due to comorbidities. Endoscopic ultrasound-guided gastroenterostomy (EUS-GE) using lumen-apposing metal stents (LAMSs) has emerged as an effective minimally invasive alternative, with high technical and clinical success and durable luminal patency 1 2 3 .

**Fig. 1:**
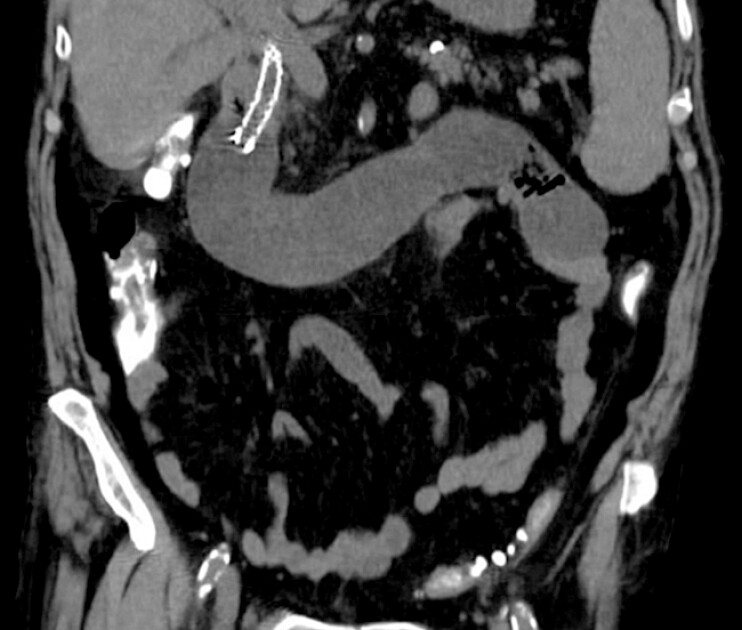
A coronal CT section demonstrating afferent loop obstruction with upstream small bowel dilation. CT, computed tomography.

**Fig. 2:**
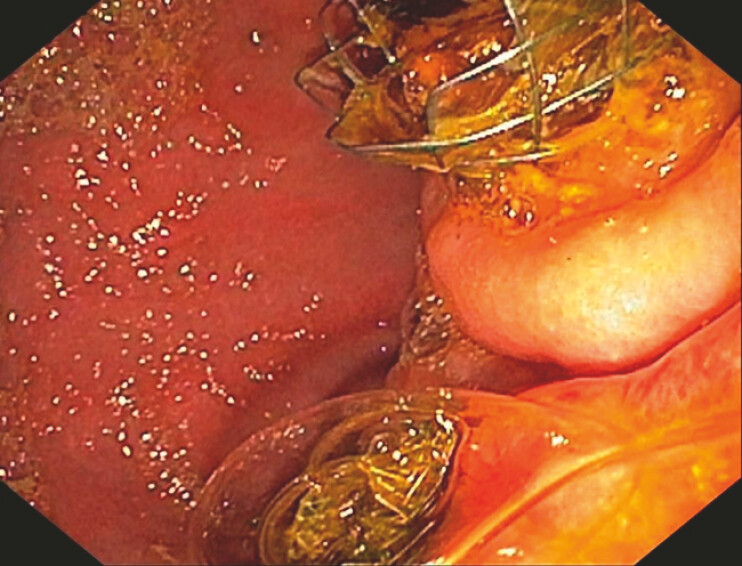
An endoscopic view of the stomach showing two lumen-apposing metal stents (LAMSs) creating gastroenteric anastomoses.

A 79-year-old man with severe chronic obstructive pulmonary disease (stage IV), peripheral arterial disease, and advanced alcohol-related liver disease was admitted for jaundice. Contrast-enhanced computed tomography and endoscopic ultrasound with fine-needle biopsy revealed unresectable advanced hilar cholangiocarcinoma (uT4N1M0). After multidisciplinary discussion, biliary drainage was achieved by endoscopic retrograde cholangiopancreatography with the placement of an uncovered biliary self-expandable metal stent (80 × 10 mm), followed by chemotherapy with gemcitabine and cisplatin. Approximately 1 year later, the patient presented with abdominal pain, nausea, and vomiting. Computed tomography demonstrated malignant duodenal obstruction, which was treated with EUS-GE using a 20 × 10 mm lumen-apposing metal stent. Six months later, recurrent symptoms occurred due to stenosis of the efferent jejunal limb distal to the anastomosis. An enteral metal stent (22 × 60 mm) was deployed through the lumen-apposing metal stent. Two months later, the patient was readmitted for abdominal pain and cholangitis. Computed tomography revealed afferent loop obstruction caused by stent migration, with upstream dilation ( Fig. 1 ). After multidisciplinary evaluation, a second endoscopic ultrasound-guided gastrojejunostomy was performed ( Fig. 2 ), achieving a rapid resolution of cholangitis in combination with antibiotic therapy ( Video 1 ). Oral intake was gradually reintroduced and well tolerated, with improvement in abdominal symptoms and nutritional status. The patient was discharged and continued palliative care without further complications.

EUS-guided placement of a second 10 × 10 mm LAMS to relieve afferent limb obstruction caused by a malpositioned SEMS, restoring effective enteric and biliary drainage. EUS, endoscopic ultrasound; LAMS, lumen-apposing metal stent; SEMS, self-expandable metal stent.Video 1

Endoscopy_UCTN_Code_TTT_1AS_2AK
